# Molecular Landscape of the Ribosome Pre-initiation Complex during mRNA Scanning: Structural Role for eIF3c and Its Control by eIF5

**DOI:** 10.1016/j.celrep.2024.114404

**Published:** 2024-06-14

**Authors:** Eiji Obayashi, Rafael E. Luna, Takashi Nagata, Pilar Martin-Marcos, Hiroyuki Hiraishi, Chingakham Ranjit Singh, Jan Peter Erzberger, Fan Zhang, Haribabu Arthanari, Jacob Morris, Riccardo Pellarin, Chelsea Moore, Ian Harmon, Evangelos Papadopoulos, Hisashi Yoshida, Mahmoud L. Nasr, Satoru Unzai, Bryttney Thompson, Eric Aube, Samantha Hustak, Florian Stengel, Eddie Dagraca, Asokan Ananbandam, Philip Gao, Takeshi Urano, Alan G. Hinnebusch, Gerhard Wagner, Katsura Asano

(Cell Reports *18*, 2651–2663; March 14, 2017)

As this article was published on March 14, 2017, the first name of Bryttney Thompson was misspelled “Brytteny.” Due to the age of the article, we are unable to correct the typo in the paper’s author list directly, but we are publishing this correction notice to acknowledge the error. The authors regret the mistake.

